# The neuroprotection of deproteinized calf blood extractives injection against Alzheimer's disease via regulation of Nrf-2 signaling

**DOI:** 10.18632/aging.202776

**Published:** 2021-03-26

**Authors:** Yidi Qu, Wenqi Wang, Tianrui Chen, Yumin Yang, Yizhi Zhang, Di Wang

**Affiliations:** 1 School of Life Sciences, Jilin University, Changchun 130012, China; 2 Department of Neurology, The Second Hospital of Jilin University, Jilin University, Changchun 130041, China; 3 Department of Bone Tumor Surgery, Changzheng Hospital, Second Military Medical University, Shanghai 200003, China

**Keywords:** Alzheimer’s disease, deproteinized calf blood extractives injection, apoptosis, oxidative stress, Nrf-2 pathway

## Abstract

Alzheimer’s disease (AD) is characterized by cognitive decline due to the accumulation of extracellular β-amyloid (Aβ) plaques and neurofibrillary tangles in the brain, which impair glutamate (Glu) metabolism. Deproteinized Calf Blood Extractive Injection (DCBEI) is a biopharmaceutical that contains 17 types of amino acids and 5 types of nucleotides. In this study, we found that DCBEI pretreatment reduced L-Glu-dependent neuroexcitation toxicity by maintaining normal mitochondrial function in HT22 cells. DCBEI treatment also reduced the expression of pro-apoptosis proteins and increased the expression of anti-apoptosis proteins. Furthermore, DCBEI attenuated AD-like behaviors (detected via the Morris water maze test) in B6C3-Tg (APPswePSEN1dE9)/Nju double transgenic (APP/PS1) mice; this effect was associated with a reduction in the amount of Aβ and neurofibrillary tangle deposition and the concomitant reduction of phospho-Tau in the hippocampus. Metabonomic profiling revealed that DCBEI regulated the level of neurotransmitters in the hippocampus of APP/PS1 mice. Label-free proteomics revealed that DCBEI regulated the expression of Nrf-2 and its downstream targets, as well as the levels of phospho-protein kinase B and mitogen-activated protein kinase. Together, these data show that DCBEI can ameliorate AD symptoms by upregulating Nrf2-mediated antioxidative pathways and thus preventing mitochondrial apoptosis.

## INTRODUCTION

Deproteinized Calf Blood Extractive Injection (DCBEI) is a sterile solution that contains more than 200 bioactive constituents including inorganics and small molecule organics; it is derived from fresh calf blood by deproteinization, ultrafiltration and incrassation [1]. DCBEI is approved for clinical use in some Asian and Eastern European countries. The main disease areas in which DCBEI is used are brain ischemia, dysneuria and craniocerebral trauma, peripheral arterial disease and diabetic polyneuropathy. DCBEI reduces pathophysiology in the brain by improving oxygen uptake and utilization, thereby enhancing oxidative metabolism; the treatment also enhances glucose uptake and mitochondrial energy metabolism [2]. DCBEI reduces the level of reactive oxygen species (ROS), which prevents neurons from undergoing apoptosis [2]. In rats with transient global cerebral ischemia, DCBEI improves spatial learning and memory [1]. To date, however, the therapeutic effects of DCBEI in Alzheimer's disease (AD) have not been systematically reported.

China is moving toward an aging society. According to statistics, there were 10 million AD patients in China by 2018, which accounted for one fifth of all AD patients worldwide. AD is associated with unavoidable societal problems such as the economic and healthcare burdens associated with care of the elderly. Indeed, the cost of caring for those with AD has surpassed the cost of managing heart disease, cancer and stroke [3]. The major clinical feature of AD is progressive cognitive decline. However, the underlying pathogenesis is poorly understood because multiple genetic and environmental factors contribute to the disease. Cognitive decline is associated with the accumulation of extracellular amyloid-β (Aβ) plaques and neurofibrillary tangles in the brain that are precipitated by hyperphosphorylation of the Tau protein [4]. Increased levels of Aβ1-40 and Aβ1-42 are associated with oxidative stress in the hippocampus [5]. The accumulation of these peptides occurs earlier in the AD brain and before the appearance of the amyloid plaques; therefore, these peptides are implicated in AD pathology [6]. Aβ deposited in plaques exerts neurotoxic effects in a variety of ways, including the disruption of mitochondrial function by binding to alcohol dehydrogenase [7], inhibition of anti-apoptotic insulin signaling [8], and the physical disruption of functional connections between neurons [9]. Induction of oxidative stress may trigger Aβ deposition [10] and Tau phosphorylation [11]. Thus, preventive strategies that control oxidative stress and target neuronal function may prevent AD.

Glutamate (Glu) is an excitatory neurotransmitter in the brain [12], and an excessive level of Glu is associated with ROS accumulation and increased calcium influx [13]. Glu metabolism is impaired in AD patients, and its accumulation damages neurons via excitotoxicity [14]. L-glutamate (L-Glu)-induced neurotoxicity in HT22 cells is an established model that recapitulates the oxidative stress and mitochondrion-dependent apoptosis that is observed in AD neurons [13, 15]. β-amyloid precursor protein (β-APP) and Presenilin 1 (PS1) play important roles in AD [16], as each of these proteins significantly accelerates the rate of decline in cognitive function, which in turn leads to an accelerated AD clinical course [17].

In this study, we systematically investigated the neuroprotective effects of DCBEI using a cellular model of glutamate-induced excitotoxicity and APP/PS1 mice as an AD model. Based on the results of our proteomic and metabonomic studies, we conclude that DCBEI-dependent neuroprotection is due to its ability to attenuate mitochondria-driven oxidative stress. Our data suggest that DCBEI could be used as an adjuvant agent for AD therapy in the clinical setting.

## RESULTS

### The detection of DCBEI compositions

Seventeen amino acids were identified in DCBEI, the most abundant of which were phenylalanine (15.37 g/kg), histidine (10.21 g/kg) and glutamic acid (10.16 g/kg) (Table 1). Of the five types of nucleotide that were detected, the levels of hypoxanthine nucleotide (9.19 mg/100 g) and uridylic acid (9.04 mg/100 g) were highest (Table 1). The ribose content of DBCEI was 140.07 mg/kg, and the molecular weight distribution of DBCEI was predominantly between 0 and 500 kDa (93.92%) (Table 1).

**Table 1:** The composition of DCBEI.

	**Compounds**	**Contents**	**Compounds**	**Contents**	**Compounds**	**Contents**
Amino acid (g/kg)	Aspartic acid (Asp)	0.02	Serine (Ser)	0.24	Phenylalanine (Phe)	15.37
	L-Threonine (Thr)	0.17	Valine (Val)	0.97	Valine (Lys)	0.93
	Serine (Ser)	0.24	Methionine (Met)	3.13	Histidine (His)	10.21
	Glutamic acid (Glu)	10.16	Isoleucine (Ile)	1.12	Arginine (Arg)	2.46
	Glycine (Gly)	0.56	Leucine (Leu)	2.21	Proline (Pro)	6.32
	Alanine (Ala)	1.81	Tyrosine (Tyr)	0.61		
Nucleotide (mg/ 100 g)	Cytidylic acid (CMP)	0.91	Hypoxanthine nucleotide (IMP)	9.19	Uridylic acid (UMP)	9.04
	Guanine (GMP)	0.98	adenylic acid (AMP)	2.52		
Ribose (mg/kg)		140.07				
Molecular weight distribution (%)	<100	42.08	100~300	36.17	300~500	15.67
	>500	6.08				

### DCBEI protects HT22 cells against mitochondrial apoptosis

**Figure 1:**
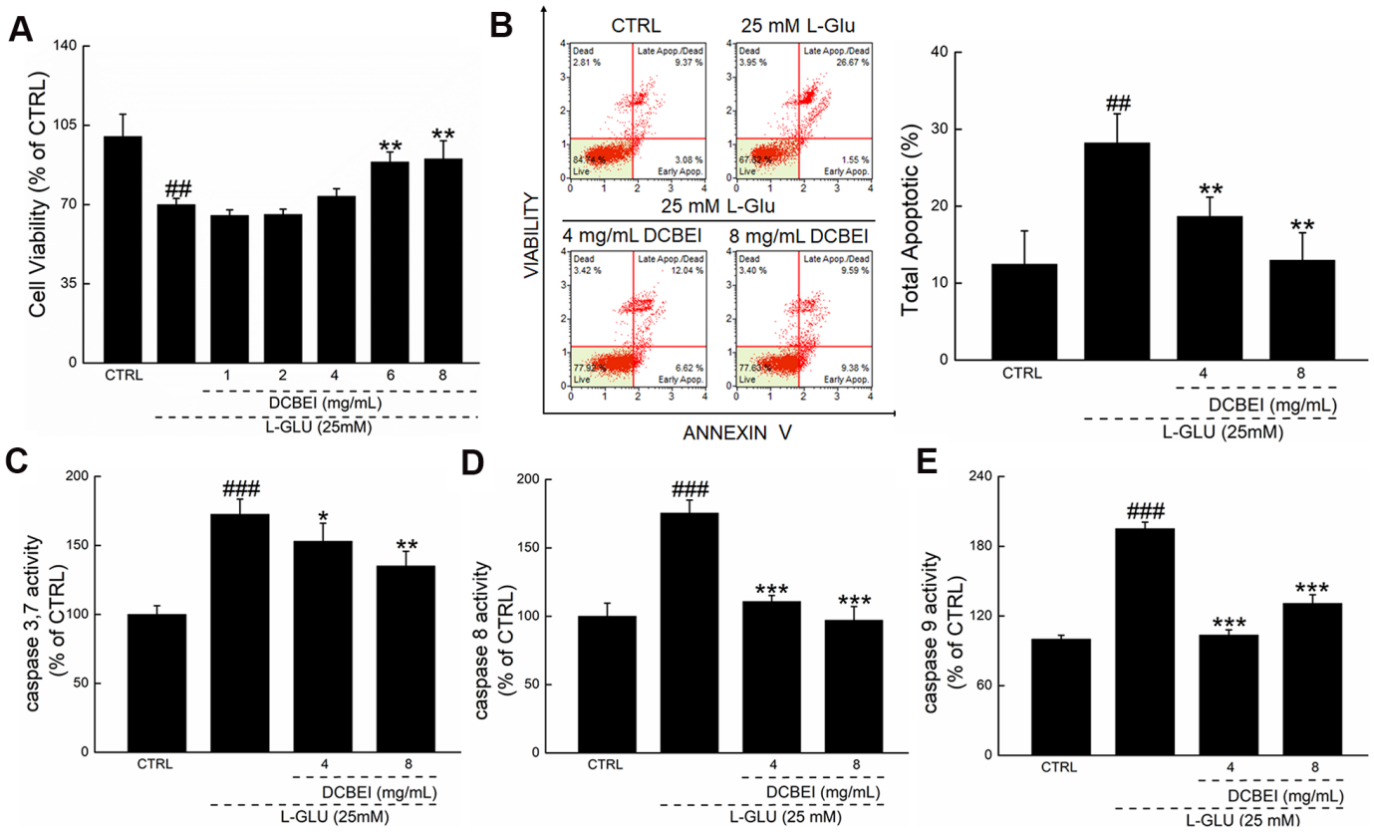
**DCBEI protects HT22 cells against L-Glu-induced damage.** HT22 cells were pre-incubated with DCBEI (4 mg/mL or 8 mg/mL) for 3 h, and co-treated with L-Glu for a further 24 h. (**A**) DCBEI increased cell viability. (**B**) DCBEI inhibited apoptosis. DCBEI attenuated the activation of caspase-3/7 (**C**), caspase-8 (**D**) and caspase-9 (**E**). Data are expressed as a percentage of corresponding control cells and means ± S.D. (n = 6). ## *P* < 0.01 and ### *P* < 0.001 vs. CTRL, * *P* < 0.05, ** *P* < 0.01 and *** *P* < 0.001 vs. L-Glu-treated cells.

**Figure 2:**
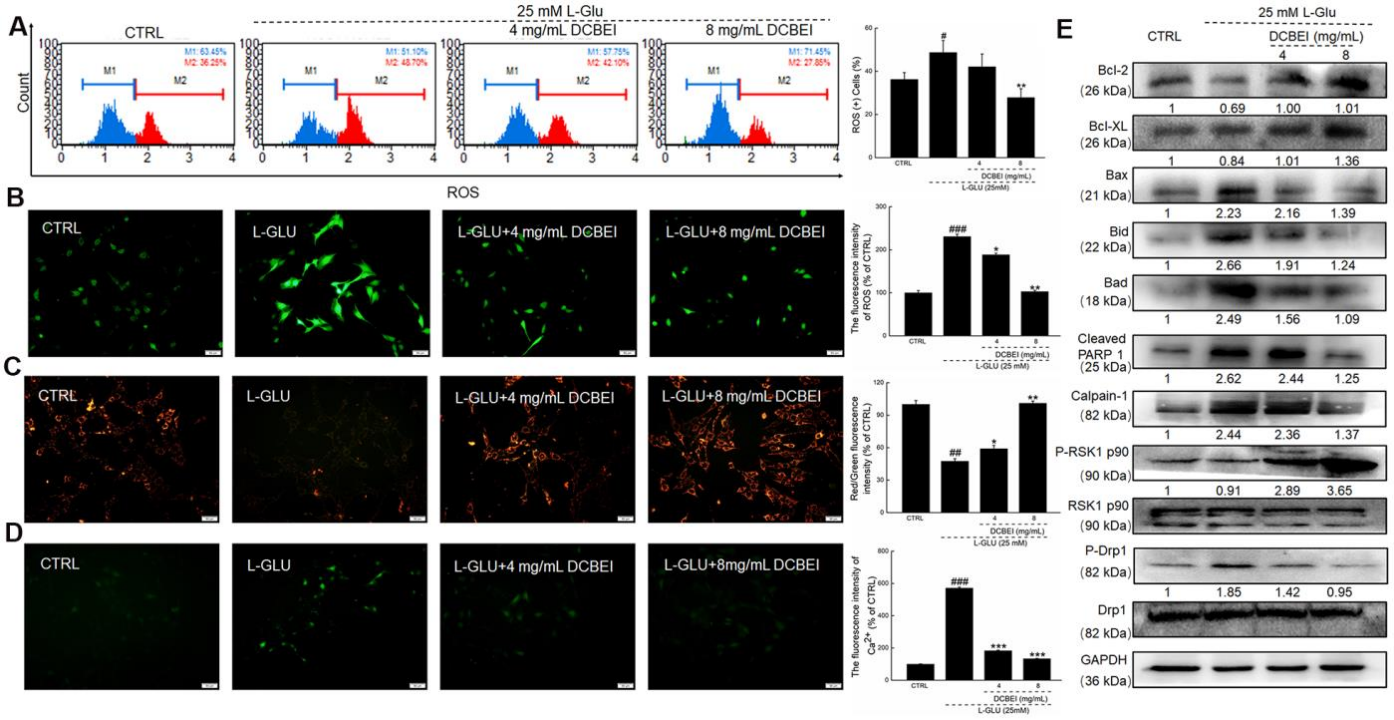
**HT22 cells were pre-incubated with DCBEI (4 mg/mL or 8 mg/mL) for 3 h, and co-treated with L-Glu for a further 24 h.** (**A**) DCBEI reduced the intracellular ROS activity in L-Glu-treated HT22 cells (n = 3). (**B**) DCBEI reduced L-Glu-induced ROS production (200×) (Scale bar: 50 μm) (n = 3). (**C**) DCBEI attenuated L-Glu-induced MMP dissipation (200×) (Scale bar: 50 μm) (n = 3). (**D**) Fluo 4-AM staining indicated that DCBEI suppressed L-Glu-induced Ca^2+^ increases (200×) (Scale bar: 50 μm) (n = 3). (**E**) DCBEI regulated the expression of proteins related to mitochondrial function. DCBEI increased the levels of anti-apoptotic proteins (Bcl-2, Bcl-xL and P-RSK1 p90) and reduced the levels of pro-apoptotic proteins (Bax, Bid, Bad, cleaved PARP-1, Calpain-1 and p-Drp1) in L-Glu-treated HT22 cells. Quantification data were normalized to GAPDH and the corresponding total proteins, and reported as fold change relative to CTRL (n = 3). Data are expressed as a percentage of corresponding control cells and means ± S.D. # *P* < 0.05, ## *P* < 0.01 and ### *P* < 0.001 vs. CTRL, * *P* < 0.05, ** *P* < 0.01 and *** *P* < 0.001 vs. L-Glu-treated cells.

L-Glu reduced the viability of HT22 cells by 30.1%, whereas DCBEI improved >20% cell viability, especially at concentrations of 6 mg/mL to 8 mg/mL (*p.* < 0.01; Figure 1A). Compared to cells treated only with L-Glu, those co-treated with DCBEI showed suppressed cell apoptosis rate by >9.5% (*p.* < 0.01, Figure 1B) and reduced caspase-3/7 activity by 37.5% (*p.* < 0.01) (Figure 1C), caspase-8 activity by 78.3% (*p.* < 0.001) (Figure 1D) and caspase-9 activity by 64.2% (*p.* < 0.001) (Figure 1E). DCBEI at concentrations of 4 mg/mL and 8 mg/mL strongly suppressed the accumulation of ROS, as indicated by the reduced red fluorescence intensity (Figure 2A) and green fluorescence intensity (Figure 2B), and prevented the dissipation of MMP, as indicated by the reduced green/red fluorescence ratio (Figure 2C). DCBEI also inhibited Ca^2+^ influx in L-Glu-treated HT22 cells, as indicated by the reduced green fluorescence intensity of the reporter (Figure 2D). Additionally, DCBEI suppressed the induction of pro-apoptotic proteins including Bax, Bid, Bad, cleaved PARP-1, Calpain-1 and phospho-Drp1, and increased the levels of anti-apoptotic proteins including Bcl-2, Bcl-XL and phospho-RSK1 p90 in L-Glu-treated HT22 cells (Figure 2E).

Aβ1-42 treatment of U251 significantly reduced survival due to the induction of apoptosis; this effect was reversed by treatment with 4 mg/mL to mg/mL DCBEI (*P* < 0.05). Compared to cells treated only with Aβ1-42, those co-treated with DCBEI showed suppressed cell apoptosis rate (*P* < 0.01, Supplementary Figure 1).

### DCBEI alleviated the AD-like symptoms in APP/PS1 mice

**Figure 3:**
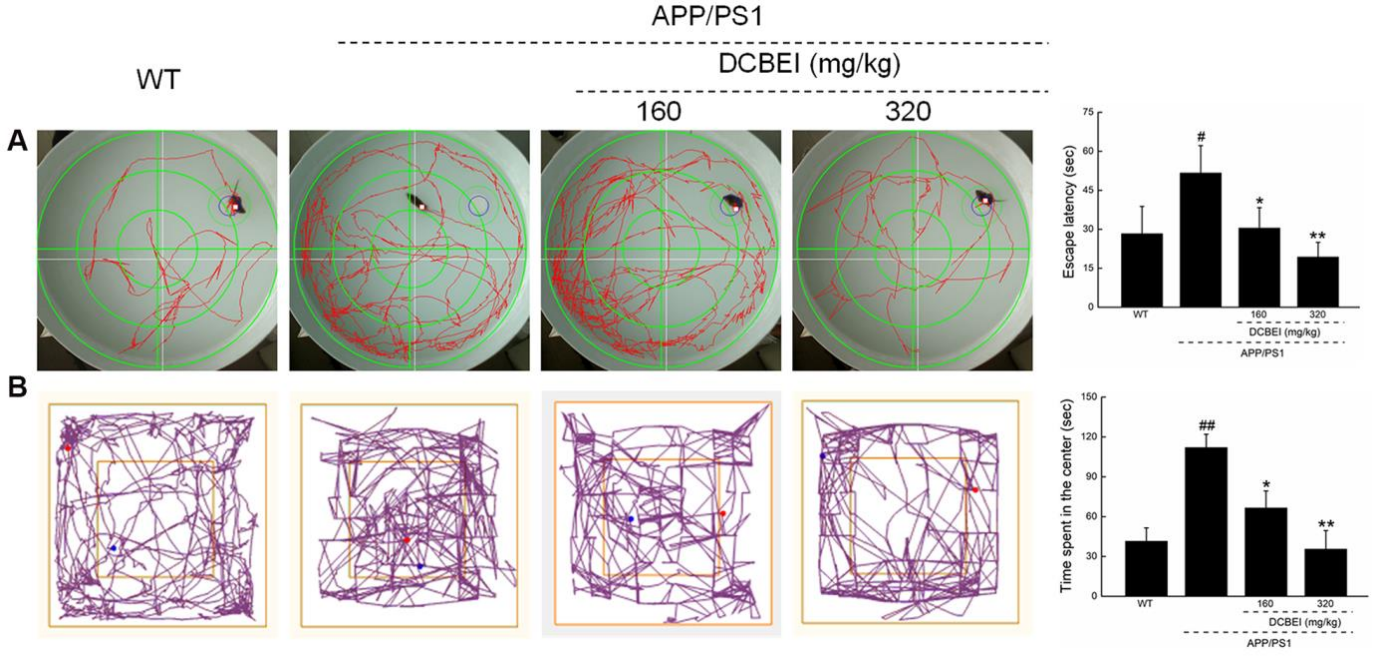
**DCBEI improves the behavioral performance of APP/PS1 mice.** Compared with non-treated APP/PS1 mice, a 28-day course of DCBEI (**A**) reduced the escape latency time in the Morris water maze test, (**B**) decreased the time taken for mice to enter the central area in an open field test. Data are expressed as means ± S.D. (n = 10). # *P* < 0.05 and ## *P* < 0.01 vs. WT, * *P* < 0.05 and ** *P* < 0.01 vs. APP/PS1 mice.

In the behavioral tests, administration of DCBEI for 28-days reduced the escape latency that APP/PS1 mice in the Morris water maze test (30.50 ± 7.84 s [DCBEI at 160 mg/kg, *p* < 0.05] and 19.33 ± 5.64 s [DCBEI at 320 mg/kg, *p* < 0.01] versus 51.67 ± 10.54 s [APP/PS1mice]; Figure 3A). It also reduced the time of mice took to enter the central area in the open field test (66.53 ± 12.79 s [DCBEI at 160 mg/kg, *p* < 0.05] and 35.58 ± 13.94 s [DCBEI at 320 mg/kg, *p* < 0.01] versus 112.07 ± 9.94 s [APP/PS1mice]; Figure 3B).

**Figure 4:**
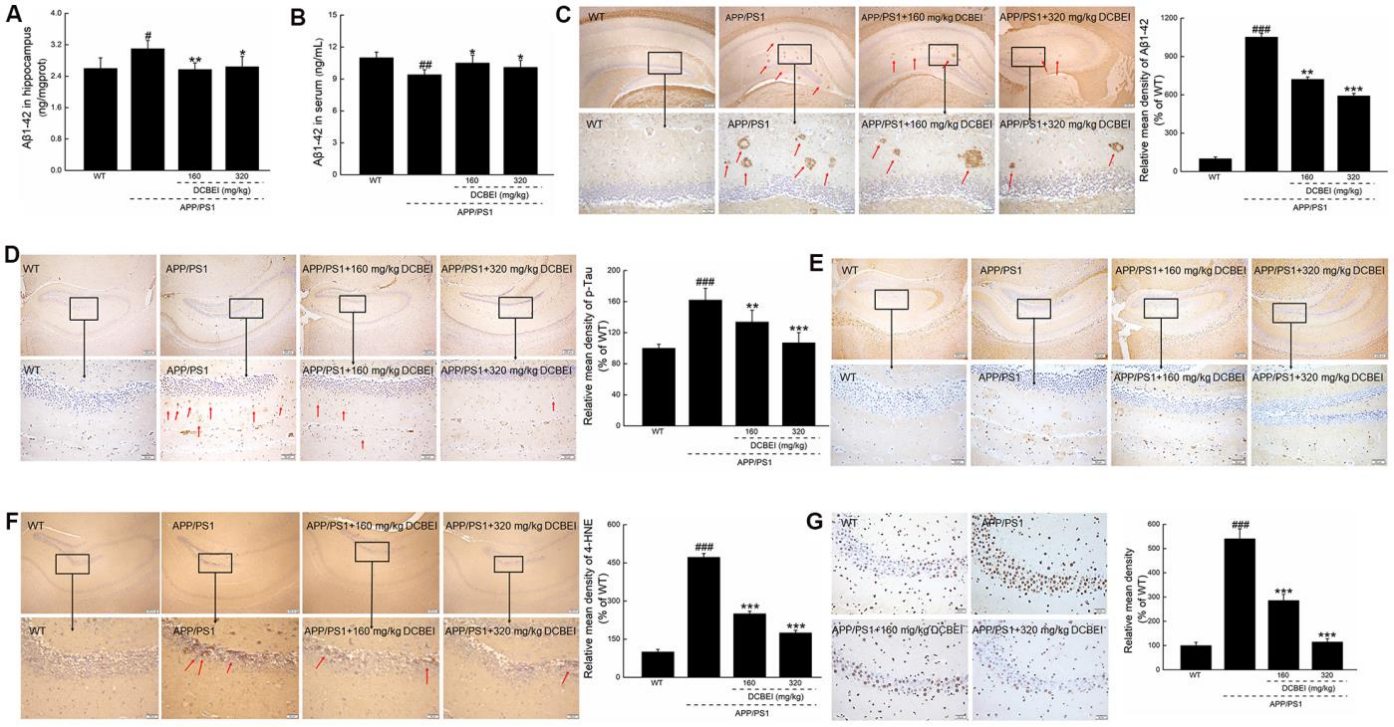
**DCBEI attenuates pathology in the brains of APP/PS1 mice.** DCBEI (**A**) reduced the expression of Aβ1-42 in the hippocampus (n = 10), and (**B**) increased the levels of Aβ1-42 in the serum (n = 10). Data are expressed as means ± S.D. (n = 10). # *P* < 0.05 and ## *P* < 0.01 vs. WT, * *P* < 0.05 and ** *P* < 0.01 vs. APP/PS1 mice. (**C**) DCBEI reduced the level of Aβ in the hippocampus (n = 3). (**D**) DCBEI attenuated the increase in p-Tau levels in the hippocampus (n = 3), (**E**) but did not affect the levels of total Tau (n = 3). (**F**) DCBEI significantly reduced the levels of 4-HNE in the brains of APP/PS1 mice (n = 3) (40×) (Scale bar: 200 μm) (200×) (Scale bar: 50 μm). (**G**) TUNEL staining shows that a 28-day course of DCBEI significantly reduces apoptosis (n = 3) (200×) (Scale bar: 50 μm).

As Aβ1-42 forms the core of amyloid plaques, it is heavily implicated in the pathology associated with plaque deposition. Compared with non-treated APP/PS1 mice, treatment with 160 mg/kg and 320 mg/kg DCBEI for 28 days reduced hippocampal Aβ1-42 concentrations by 17.1% and 14.8%, respectively (*p* < 0.01) (Figure 4A). There were concomitant increases of 11.7% and 7.4% in the serum concentration of Aβ1-42 with 160 mg/kg and 320 mg/kg DCBEI, respectively (*p* < 0.05) (Figure 4B).

The DCBEI-dependent reduction of Aβ1-42 deposition—especially in the hippocampus—was further confirmed by pathologic analysis (Figure 4C). Compared with non-treated APP/PS1 mice, DCBEI suppressed the induction of phospho-Tau (p-Tau) in the hippocampus (Figure 4D), but had no effect on the levels of total Tau (Figure 4E). DCBEI strongly reduced the levels of 4 Hydroxynonenal (4-HNE), a key mediator of oxidative stress-induced damage, in the hippocampus (Figure 4F). DCBEI also significantly reduced the amount of TUNEL-positive apoptotic neurons in the brains of APP/PS1 mice (Figure 4G). DCBEI did not induce significant gross pathology in the brain, spleen or kidney (Supplementary Figure 2), suggesting that it could be used safely.

### The regulation of DCBE on neurotransmitters of APP/PS1 mice

**Figure 5:**
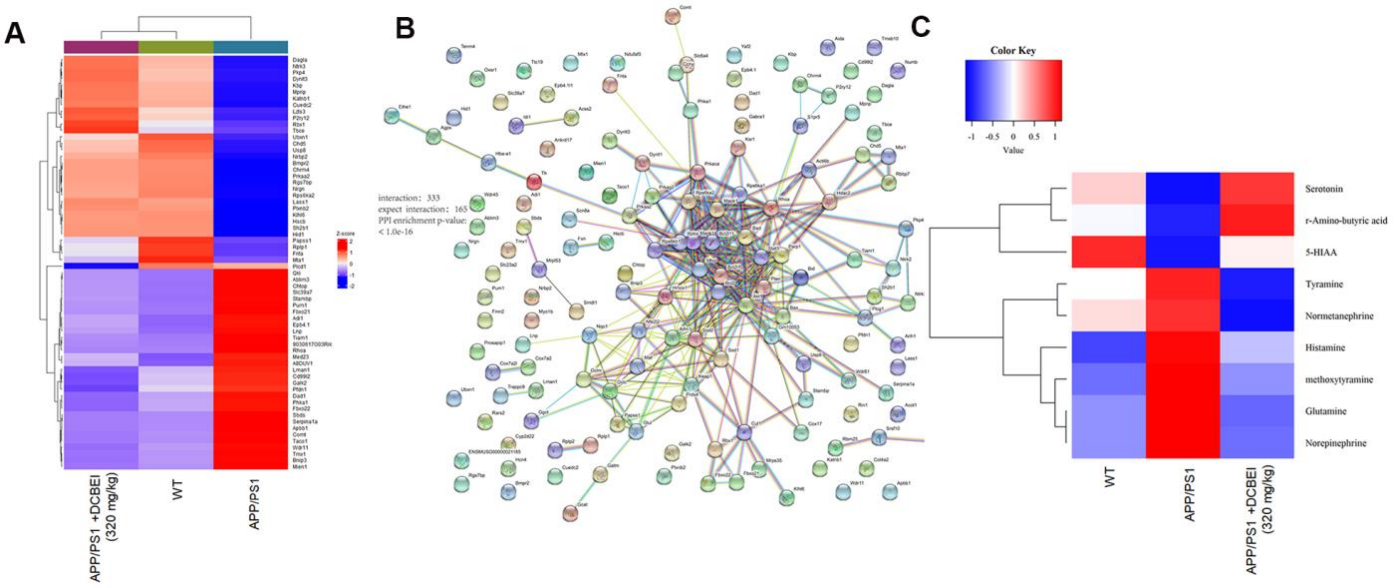
**Proteomic and metabonomic profiling of the effects of DCBEI in APP/PS1 mice.** (**A**) Heat map of differentially expressed factors (n = 3). (**B**) The relationship between proteins processed through STRINGdb. (**C**) Serum neurotransmitter levels were quantified using HPLC-MS/MS. Significant differences in the levels of nine neurotransmitters (*R*-amino-butyric acid, norepinephrine, 5-HIAA, serotonin, methoxytyramine, histamine, tyramine, glutamine and normetanephrine) was observed (n = 3).

The serum content of nine neurotransmitters including r-Amino-butyric acid (GABA), Norepinephrine (NA), 5-hydroxyindoleacetic acid (5-HIAA), serotonin (5-HT), Histamine (HIS), glutamine (Gln) and isoprenaline hydrochloride (iso-Hyd) was quantified using HPLC-MS/MS (Figure 5C and Supplementary Figure 3). Based on this initial quantification, Enzyme-linked immunosorbent assay (ELISA) was used to quantify the levels of seven of these neurotransmitters in whole brain tissues. Compared with non-treated APP/PS1 mice, DCBEI-treated mice (160 mg/kg and 320 mg/kg) showed enhanced levels of GABA (*p* < 0.01), iso-Hyd (*p* < 0.001), 5-HT (*p* < 0.01), NA (*p* < 0.001), HIS (*p* < 0.001) and 5-HIAA (*p* < 0.01) that were >71.4%, >73.9%, >63.9%, >59.3%, >7 9.0%, and >96.6%, respectively (Table 2).

**Table 2:** The effects of DCBEI on the levels of neurotransmitters in hippocampus of APP/PS1 mice.

	**WT**	**APP/PS1**	**APP/PS1**	
			**DCBEI (mg/kg)**	
			**160**	**320**
Ach (×10^-2^) (pmoL/mgprot)	1.7±0.2	1.3±0.2^#^	2.2±0.4^*^	2.1±0.5^*^
AChE (×10^-2^) (nmoL/mgprot)	6.1±0.9	8.2±0.9^#^	7.6±0.5	7.8±1.2
ChAT (×10^-2^) (pmoL/mgprot)	2.3±0.2	2.4±0.4	2.6±0.5	5.9±0.6^**^
Gln (μmol/mgprot)	0.5±0.2	0.8±0.2^#^	0.6±0.1	0.7±0.2
GABA (×10^-3^, μmol/mgprot)	3.1±0.5	2.4±0.4^#^	4.1±0.7^**^	4.5±0.8^***^
iso-Hyd (μg/mgprot)	657.7±118.2	492.8±87.8^##^	871.1±139.0^***^	988.8±198.5^***^
5-HT (ng/mgprot)	114.0±10.6	90.1±16.1^##^	151.9±22.2^***^	181.4±57.9^**^
NA (ng/mgprot)	3.6±0.5	2.4±0.3^###^	3.8±0.5^**^	4.5±0.7^***^
HIS (ng/mgprot)	7.1±0.4	5.4±0.8^##^	9.7±1.4^***^	10.9±1.2^***^
5-HIAA (ng/mgprot)	9.9±2	7.6±1.1^#^	15.0±2.1^***^	18.2±5.3^**^

All date is expressed as mean ± S.D. (n=10). ^#^*P* < 0.05, ^##^*P* < 0.01 and ^###^*P* < 0.001 compared with WT group; ^*^P < 0.05, ^**^P < 0.01 and ^***^P < 0.001 compared with APP/PS1 group.

### The regulation of DCBEI on Nuclear factor-erythroid 2 related factor 2 (Nrf-2) signaling

Proteomics revealed that 362 proteins were differentially expressed between WT and APP/PS1 mice, and 309 proteins were differentially expressed between DCBEI-treated and untreated APP/PS1 mice (Supplementary Figure 4). After applying cut-off values of A/B > 1.5 or A/B < 0.66, we observed that 64 proteins were differentially expressed following DCBEI treatment (32 were upregulated and 32 were downregulated; Figure 5A). Gene Ontology analysis indicated an enrichment of mitochondrial proteins, suggesting that DCBEI-mediated neuroprotection is linked to the regulation of apoptosis and oxidative stress (Supplementary Figure 5). We next used STRINGdb to analyze interactions between proteins that were differentially expressed between experimental groups; this revealed 333 enriched interactions and 165 expected interactions (Figure 5B).

Liquid chromatograph-mass spectrometer/mass spectrometer (LC-MS/MS) analysis revealed changes in the levels of anti- and pro-oxidative factors in the serum and hippocampus of APP/PS1 mice. Compared with vehicle-treated APP/PS1 mice, DCBEI-treated mice showed increased concentration of catalase (CAT, *p* < 0.05) and reduced levels of ROS (*p* < 0.05) in the serum and hippocampus, enhanced levels of glutathione peroxidase (GSH-Px, *p* < 0.05) and superoxide dismutase (SOD, *p* < 0.05) in the hippocampus, and reduced levels of malondialdehyde (MDA, *p* < 0.05) in the serum (Table 3). The effect was particularly striking at the highest DCBEI dose (320 mg/kg).

**Table 3:** The effects of DCBEI on the levels of oxidative stress related factors in serum and hippocampus of APP/PS1 mice.

		**WT**	**APP/PS1**	**APP/PS1**	
				**DCBEI (mg/kg)**	
				**160**	**320**
	SOD (pg/mL)	50.3±1.98	41.3±1.1	42.0±3.3	42.8±2.9
Serum	MDA (pmoL/mL)	1.9±0.15	2.6±0.3^#^	1.9±0.2^*^	2.0±0.2^*^
	CAT (pg/mL)	28.7±1.3	25.2±1.2	26.7±1.9	31.4±2.7^*^
	GSH-Px (pg/mL)	89.9±6.6	83.3±5.8	88.5±10.6	82.6±8.8
	ROS (U/mL)	387.2±19.7	438.0±13.4^#^	384.8±12.7^*^	394.5±25.7^*^
Hippocampus	SOD (pg/mgprot)	18.3±2.6	13.6±1.2^#^	17.1±1.6^*^	17.9±1.6^*^
	MDA (×10^-2^, pmoL/mgprot)	36.8±2.7	44.4±2.7	44.3±7.3	44.2±3.1
	CAT (pg/mgprot)	9.5±1.39	7.6±0.733^#^	8.5±0.9^*^	9.4±1.2^*^
	GSH-Px (pg/mgprot)	34.9±3.0	34.2±2.8	42.4±3.4^*^	39.0±3.9^*^
	ROS (U/mgprot)	133.6±9.4	136.7±8.3	106.9±12.8^*^	106.2±11.2^*^

All date is expressed as mean ± S.D. (n=10). ^#^*P* < 0.05 compared with WT group; ^*^P < 0.05 compared with APP/PS1 group.

**Figure 6:**
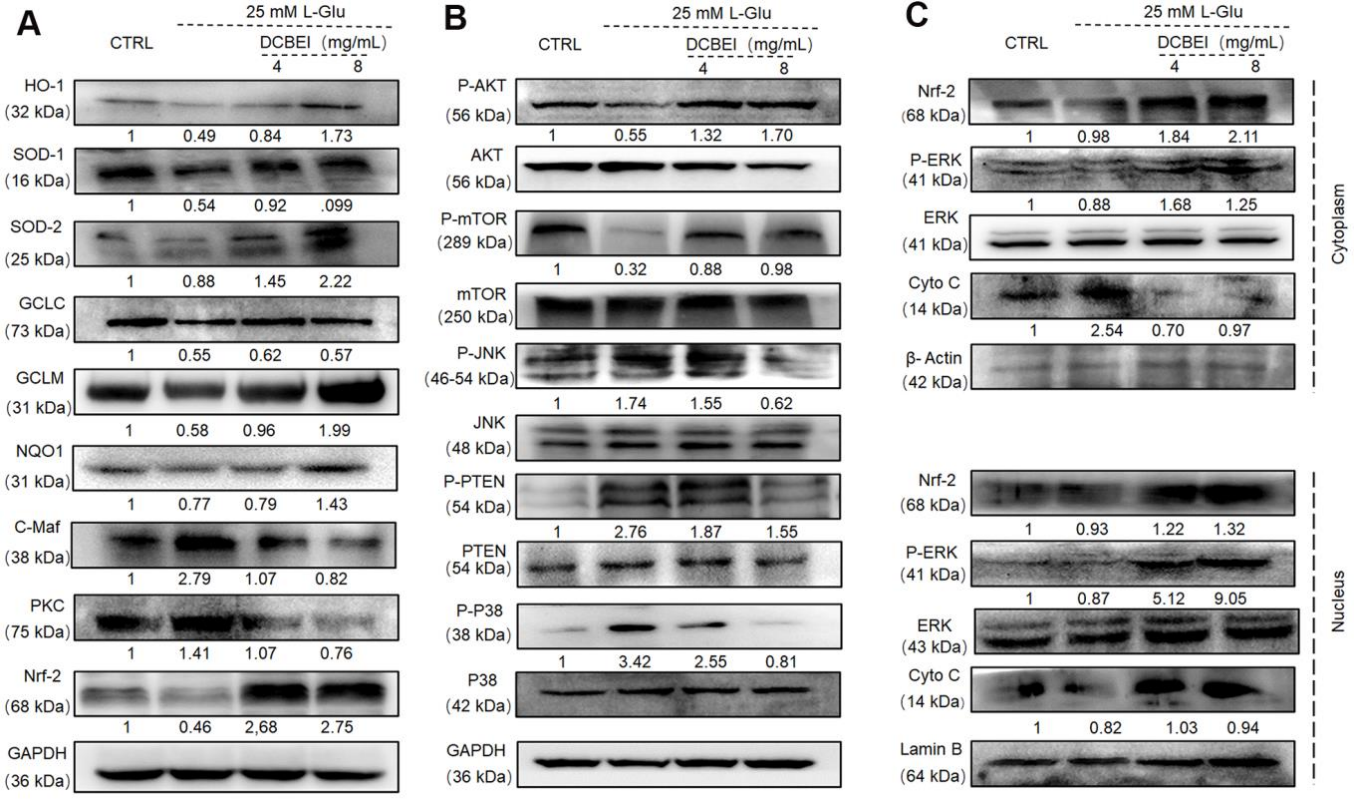
**DCBEI ameliorates AD symptoms by reducing apoptosis, attenuating oxidative stress, and regulating neurotransmitter levels via Nrf2 signaling.** (**A**) In L-Glu-exposed HT22 cells, DCBEI increased the expression of Nrf-2 and its downstream targets including HO-1, SOD-1, SOD-2, GCLC, GCLM and NQO1, and reduced the expression levels of C-Maf. (**B**) Analysis of MAPKs and Akt in L-Glu-treated HT22 cells. DCBEI increased the expression of p-AKT and p-mTOR, and reduced the expression of p-JNK, p-PTEN and p-P38 in L-Glu-treated HT22 cells. (**C**) DCBEI increases p-ERK levels and causes a redistribution of p-ERK, Nrf-2, and Cyto C from the cytoplasm to the nucleus. Quantification data were normalized to GAPDH and the corresponding total proteins, and are reported as fold change relative to CTRL (n = 3).

Following L-Glu exposure, Nrf-2 and its downstream targets (including HO-1, SOD-1, SOD-2, GCLC, GCLM and NQO1) were downregulated, whereas C-Maf and PKC were upregulated. These effects were reversed by a 3-h preincubation with DCBEI (Figure 6A). Compared with cells treated with only L-Glu, those pre-treated with DCBEI showed enhanced phosphorylation of AKT and mTOR and reduced phosphorylation of JNK, PTEN and P38 (Figure 6B). Additionally, DCBEI increased the levels of nuclear CytoC and the nuclear translocation of Nrf-2 and phospho-ERK in L-Glu-treated HT22 cells (Figure 6C).

## DISCUSSION

DCBEI, which is derived from fresh calf blood via deproteinization, ultrafiltration and incrassation, has been used in Chinese clinics (SFDA approval NO. H20060121) for several years. In the present study, we found that DCBEI contains 17 amino acids, 5 nucleotides and abundant levels of ribose, which confirms its high nutritional content. The diverse components of DCBEI not only explain its non-dose-dependent effects in most of our experiments, but also suggests that it is safe to use. The latter point was also confirmed by our pathological examinations.

When hypoxia or ischemia occurs in the brain, a large amount of Glu is released, which in turn triggers the mobilization of intracellular Ca^2+^ and continuous excitation of neurons [18]. In AD, excitotoxicity due to hyperstimulation with Glu is the ultimate step prior to the onset of neuronal necrosis and apoptosis [19]. The onset of AD pathology further reduces the ability of nerve cells to absorb Glu because it leads to decreased expression of the Glu transporter (excitatory amino acid transporter); this vicious cycle exacerbates excitotoxicity [20]. Our data clearly show that in HT22 cells, DCBEI attenuates this process.

In neurodegenerative diseases, increased mitochondrial damage is a major feature of Glu-induced neurotoxicity [21]. Mitochondrial damage leads to impaired redox, reduced ATP synthesis and increased apoptosis. Excessive L-Glu stimulates the postsynaptic Glu receptor, causing Ca^2+^ influx through N-methyl-D-aspartic acid (NMDA) receptor channels, which in turn activate the opening of mitochondrial permeability transition pores [18, 22]. Pore opening triggers excessive production of reactive metabolites, such as ROS, which further perturb mitochondrial function and Mitochondrial membrane potential (MMP) dissipation via the modulation of ATP-sensitive potassium channels [23]. Phosphorylated RSK1 p90 can inhibit ROS activity and thereby suppress oxidative stress [24]. Pro- and anti-apoptotic Bcl-2 family members regulate the permeability of the mitochondrial membrane by activation or inhibition of the inner mitochondrial permeability transition channels, respectively [25]. Excitotoxic stimuli also cause the release of Ca^2+^, which can activate Calpain-1, a pro-apoptotic factor that acts within mitochondria [26]. The phosphorylation of Drp1 is linked to mitochondrial fragmentation and is an early marker of apoptosis [27]. Excessive ROS activates JNK and P38 kinases, which are mediators of pro-apoptotic signaling; these kinases trigger Bax accumulation and thereby sensitize cells to apoptosis. Meanwhile, ROS inhibit AKT activation by promoting the phosphatase activity of PTEN, which leads to impaired mitochondrial biosynthesis [28]. AKT activity stabilizes Nrf-2, a transcription factor that upregulates antioxidant genes and thus promotes normal mitochondrial function [29]. Consistent with the key role of antioxidants in suppressing neurotoxicity, we observed that DCBEI pretreatment promoted mitochondrial function by inhibiting oxidative stress.

Due to the obvious learning and memory dysfunction and increased accumulation of Aβ that are observed in the APP/PS1 double transgenic mice, this strain has been widely used in AD pathophysiology studies [30]. The blood-brain barrier, which strictly regulates Glu transport in non-affected individuals, is compromised in AD patients, which leads to increased brain Glu concentration [31]. The development of oxidative stress, which plays a key role in AD pathogenesis and occurs early in the course of AD onset, is related to the deposition of Aβ plaques [32]. High levels of Aβ1–40 and Aβ1–42 are associated with high levels of oxidation products in the hippocampus of AD patients [33], and Aβ and ROS synergize in a feedback loop that leads to mitochondrial dysfunction and apoptosis. Furthermore, the excessive deposition of Aβ results in deposition of Tau protein, which forms the neurofibrillary tangles that cause memory deficits and synaptic degeneration [32]. Notably, our *in vitro* experiments revealed that DCBEI can inhibit Aβ1-42-induced apoptosis. Apparently, DCBEI is neuroprotective in APP/PS1 mice because it inhibits the deposition of Aβ and Tau in the hippocampus; this in turn reduces the severity of AD-like behavior in these animals. High levels of the oxidative stress biomarker 4-HNE are present in areas of the hippocampus with high Aβ deposition [32]. The accumulation of ROS and MDA can disrupt the activities of SOD and GSH-Px, which are endogenous antioxidants that promote the removal of oxygen free radicals, and of CAT, which is a key ROS buffer [34]. The reduced Nrf-2 activity that was observed in the AD hippocampus is likely to explain the downregulation of important antioxidant enzymes (such as SOD and GSH-Px) in this brain compartment. The increased oxidative stress in AD tissue engenders an abnormal accumulation of Aβ and P-Tau, thereby creating a vicious circle [29]. Following DCBEI treatment, 4-HNE levels fell concomitantly with a drop in ROS levels in the brains of APP/PS1 mice. This translated to a reduction in the amount of apoptosis in hippocampal neurons. The interactions between neurotransmitters during hippocampal neurogenesis can affect individual emotions and behaviors, and can contribute to the risk of developing neurodegenerative disease [35]. Neuronal absorption of glutamine activates the “Glu-glutamine cycle”, which is closely linked to the levels of the inhibitory neurotransmitter GABA [36, 37]. Indeed, the neuronal hyperexcitability observed in some AD patients may be due to extremely low levels of GABA [38]. The activity of 5-HT, which is directly and indirectly regulated by other neurotransmitters including Glu and GABA, has an impact on short- and long-term memory; both these cognitive functions are strongly suppressed in AD patients [39]. NA can improve cognitive function and reduce anxiety in AD patients via attenuating Aβ1-42 cytotoxicity [40]. Increased HIS is associated with the release of acetylcholine (Ach) in the cerebral cortex, which is in turn linked to enhanced memory and learning capability in experimental animals [52]. Consistent with this finding, there is evidence of decreased HIS in the brains of AD patients. Attenuated cholinergic function in AD patients is associated with impaired memory function and cognitive decline [41]. Additionally, several neurotransmitters (including HIS) can stimulate oxidative stress by enhancing the release of ROS [42].

ROS-triggered oxidative stress activates JNK and P38 [43]. In contrast, activated Nrf-2 signaling reduces oxidative stress and thus relieves the cognitive impairment that is induced by amyloid pathology [44]. Consistent with the role of nuclear Nrf-2 in regulating amyloid-induced pathology, its levels in the hippocampus of AD mice are low [29]. In L-Glu-treated HT22 cells, DCBEI stimulated the biological activity of Nrf-2 by promoting its nuclear translocation. Nrf-2 is a positive regulator of Akt and mTOR, which act together to negatively regulate PTEN, protect mitochondria, and improve cell survival [29, 45]. According to a previous study, some of the neuroprotective effects of neurotransmitters are Nrf-2-dependent [46].

The current study has one limitation. Although DCBEI has been approved by the SFDA for clinical use in China, we were unable to identify the specific constituent that was responsible for the neuroprotective effects of this biopharmaceutical.

In conclusion, DCBEI relieves AD symptoms by promoting an Nrf-2-dependent antioxidative response, which in turn prevents mitochondrial apoptosis. Our findings provide the experimental support for the use of DCBEI as adjuvant therapy to treat AD.

## MATERIALS AND METHODS

### Analysis of DCBEI composition

As described in previous studies [47–50], we determined the molecular weight distribution and the nucleotide and ribose contents of Deproteinized Calf Blood Extractive Injection (DCBEI)(CAS: 20160803; obtained from Jilin Connell Pharmaceutical Co. LTD., Jilin, China) samples using high-performance liquid chromatography (HPLC) (E2695, Waters Co., Ltd., USA) systems and gel permeation chromatography (1515, Waters Co., Ltd., USA).

DCBEI was hydrolyzed by the addition of 6 mol/L of hydrochloric acid at 110° C for 24 h, and then dried under vacuum. Deionized water was added to prepare DCBEI test samples and amino acid standards. Seventeen distinct free amino acids were detected using the HPLC system as described in a previous study [51].

### Cell culture

HT22, a mouse hippocampal neuronal cell line (BNCC-337709) (Chinese Academy of Sciences Cell Bank, Shanghai, China), and U251, a human astroglioma cell line (No. BNCC337874), were cultured in Dulbecco’s Modified Eagle’s Media (Gibco, USA) containing 10% fetal bovine serum (Gibco, USA) supplemented with 100 units/mL penicillin and 100 μg/mL streptomycin (Invitrogen, USA) under a humidified atmosphere containing 5%/95% CO_2_/air at 37° C.

### Cell viability and caspase activity analyses

HT22 cells (6 × 10^3^ cells/100 μL) were seeded into 96-well plates, pre-treated with DCBEI at doses of 1 mg/mL to 8 mg/mL for 3 h, and then treated with L-Glu (25 mM) for another 24 h.

U251 cells (6 × 10^3^ cells/100 μL) were seeded into 96-well plates, pre-treated with DCBEI at doses of 1 mg/mL to 8 mg/mL for 3 h, and then treated with Aβ1-42 (10 μM) (purity ≥ 95%, Gill Biochemical Co., Ltd., Shanghai, China) for another 24 h [52].

A cell viability assay using 3-(4,5-dimethylthiazol-2-yl)-2,5-diphenyltetrazolium bromide (MTT) was performed as described in our previous study [53].

HT22 cells (3 × 10^5^ cells/mL) were seeded into 6-well plates, pre-treated with 4 mL or 8 mL of DCBEI for 3 h, and then treated with L-Glu (25 mM) for another 24 h. The activities of Caspase-3/7 (APT423), -8 (APT408) and -9 (APT409) were analyzed using commercial kits (Millipore, Billerica, MA, USA) according to the manufacturer’s instructions.

### Quantification of apoptosis and ROS activities via flow cytometry

HT22 cells (3 × 10^5^ cells/mL) were seeded into 6-well plates, pre-treated with DCBEI at doses of 4 mL and 8 mL for 3 h, and then treated with L-Glu (25 mM) for another 24 h.

U251 cells (3 × 10^5^ cells/mL) were seeded into 6-well plates, pre-treated with 4 mL or 8 mL of DCBEI for 3 h, and then treated with Aβ1-42 (10 μM) for another 24 h.

Cells were collected and stained with reagents of the Dead Cell Kit (MCH100105, Millipore, Billerica, MA, USA) and Oxidative Stress Kit (MCH100111, Millipore, Billerica, MA, USA) at 37° C in the dark for 15 min. Fluorescence intensity, as an indicator of apoptosis and ROS activity, was measured using a Muse™ Cell Analyzer (Millipore, Billerica, MA, USA).

### Assessment of the dissipation of MMP, and intracellular levels of ROS and Ca^2+^

HT22 cells (1 × 10^5^ cells/mL) were seeded into 6-well plates, pre-treated with 4 mL or 8 mL of DCBEI for 3 h, and then treated with L-Glu (25 mM) for another 24 h.

Treated cells were stained with 10 μM of 2,7-dichlorofluorescein diacetate (DCFH-DA; Nanjing Jiancheng Bioengineering Institute, Nanjing, China), 5 μM of Fluo-4-AM (Molecular Probes, USA), or 2 μmol/L of 5,5′,6,6′-Tetrachloro-1,1′,3,3′-tetraethylbenzimidazolylcarbocyanine iodide (JC-1, Millipore, Billerica, MA, USA) for 20 min at room temperature in the dark. Intracellular ROS and Ca^2+^ levels, which were indirectly reported by the green fluorescence, and the degree of MMP l, which was reported by the ratio of red/green fluorescence, were quantified using a Nikon TE2000 fluorescence microscope with a CCD camera (Japan) (200×).

### Western blot

HT22 cells were seeded into 6-well plates (3 × 10^5^ cells/well), pre-treated with 4 mL or 8 mL of DCBEI for 3 h, and then treated with L-Glu (25 mM) for another 24 h. The concentration of protein in cell lysates was measured using BCA protein assay kit (Millipore, Billerica, MA, USA). Sodium dodecyl sulfate polyacrylamide gel electrophoresis (10–12%) was used to separate the protein lysates (40 μg), which were transferred to polyvinylidene difluoride membranes (0.45 μm, Merck Millipore, Billerica, MA, USA). The membranes were blotted with 5% bovine serum albumin (Sigma, USA) at 4° C for 4 h, and then incubated with primary antibodies (Supplementary Table 1) at 4° C for 12 h. After washing with Tris-buffered saline-Tween-20 buffer, the membranes were incubated with horseradish peroxidase (HRP)-conjugated secondary antibody [anti-mouse (IH-0031) and anti-rabbit (IH-0011)] (Dingguo, Beijing, China) at 4° C for 4 h. An ECL kit (WBKLS0500, Millipore, Billerica, MA, USA) was used to visualize the protein bands, and the intensity of the bands was analyzed using ImageJ software (National Institutes of Health, Bethesda, MD, USA).

### Animal husbandry and drug treatment

The experiment was conducted with the approval of the Institution Animal Ethics Committee of Jilin University (License No. SY0604). Forty-five B6C3-Tg (APPswePSEN1dE9)/Nju double transgenic male mice [Genotype: (Appswe)T, (Psen1) T] (APP/PS1) (8 months old, 45–50 g) and 15 wild-type male mice [Genotype: (Appswe)W, (Psen1) W] (WT) (8 months old, 45–50 g) were purchased from Nanjing Biomedical Research Institute of Nanjing University, Jiangsu, China [SCXK (SU) 2015-0001]. All mice were raised in a room held at 23 ± 2° C with a humidity of 40–60% under a 12-h:12-h light/dark cycle; the animals were given access to water and food *ad libitum*.

APP/PS1 mice were randomly assigned to three groups (n = 15/group), and received an intraperitoneal injection of DCBEI (160 mg/kg and 320 mg/kg), or an equivalent volume of saline for 28 days. After the last behavioral test, the mice were euthanized by injection with 150 mg/kg of 1.5% pentobarbital solution. The serum together with tissues of the brain, spleen and kidney were collected for biochemical and pathological analyses.

### Behavioral tests

### Morris water maze experiment test

The long-term spatial memory and learning capabilities of mice were evaluated using the Morris water maze [54]. After 5 days of training, mice were put into the MT-200 Water Labyrinth Video Tracking Analysis System (S7200) with a circular pool (122 cm diameter, 62.5 cm deep) filled with water to a depth of 40 cm (24 ± 2° C) containing titanium dioxide; this pool was higher than the platform. The amount of time spent searching the platform over a 60-s period and the movement trajectory were recorded by a video camera.

### Open field experiment test

An open field test was used to evaluate the autonomic activities, exploration behaviors, and anxiety behaviors of experimental animals when placed in new environments [54]. The open field test apparatus (50 cm long × 50 cm wide × 50 cm deep) was protected with an isolator, and divided into a central area (25 cm long × 25 cm wide) and a surrounding area. The movements of mice were recorded for 5 min, and their trajectory was analyzed by software (Any-maze™, Stoelting Co., Chicago, IL, USA). The apparatus was cleaned between successive mice to ensure that no information from the preceding animal was present.

### Immunohistochemistry examination

Paraffin sections of tissues were deparaffinized in xylene, rehydrated in different concentrations of ethanol, and washed twice in phosphate-buffered saline solution (PBS). The tissue slides were heated with citrate buffer for 15 min to retrieve antigens, incubated with hydrogen peroxide for 12 min at room temperature to block endogenous peroxidase, and then blocked with 5% normal horse serum and 1% normal goat serum dissolved in PBS. The slides were then incubated with anti-amyloid precursor (Aβ) antibody (1:500 dilution; ab32136), anti-Tau antibody (1:750 dilution; ab32057), anti-phospho-Tau (ser404) (1:200 dilution; ab 92676) and anti-4-HNE antibody (1:200 dilution; ab46545) (Abcam, Cambridge, MA, USA) at 25° C for 1 h. The slides were stained with 3,3′-diaminobenzidine and Mayer’s hematoxylin and visualized and photographed using a microscope (40× and 200×; IX73, Olympus, Tokyo, Japan).

### TUNEL assay

Brain tissue sections were deparaffinized at 37° C, and then exposed to permeabilization reagent (Proteinase K solution) for 15 min. Sections of mouse hippocampus were labelled with the Click-it™ Plus TUNEL assay kit (C10617, Invitrogen, USA) according to the manufacturer’s instructions. The slides were stained with 3,3′-diaminobenzidine and apoptosis was then quantified using a microscope (200×; IX73, Olympus, Tokyo, Japan).

### LC-MS/MS analysis of serum neurotransmitters

Based on previous studies [55, 56], 100 μL of serum from each mouse was homogenized in 400 μL ice cold methanol (Sigma Aldrich Fluka, USA)/acetonitrile (ACN; Merck, GER) (V:V = 1:1) with 1% formic acid (Merck, GER), and the mixture was incubated for 60 min at 37° C until a liquid supernatant was obtained. After vacuum drying, the precipitate of liquid supernatant was dissolved in ACN/water (1:1, v/v) with 1% FA.

The concentrations of NA, 5-HT, and 5-HIAA, Glu, GABA, Gln, HIS, normetanephrine, tyramine and methoxytyramine were quantified using an integrated HPLC-MS/MS system. All standards were purchased from Sigma-Aldrich (USA).

### Label-free quantification (LFQ) analysis of proteins in the brains

### Preparation of protein samples

The brain tissue samples were homogenized in RIPA buffer containing protease inhibitor cocktail (Kangchen Bio-tech, Shanghai, China) and 1 mM Phenylmethylsulfonyl fluoride (PMSF), followed by 1 min of sonication. Samples were then centrifuged and the supernatant containing the whole tissue extract was retained. Protein concentration was determined using a BCA assay. Sample preparation was based on previous studies [57], and all samples were processed simultaneously. Samples were analyzed using LC-MS/MS.

### LC-MS/MS analysis

For each sample, 2 μg of peptide was separated and analyzed with a Nano-HPLC (EASY-nLC1200) coupled to a Q-Exactive mass spectrometer (Thermo Finnigan). The separation conditions used were the same as those reported in a previous study.

Data-dependent acquisition (DDA) was performed in the profile and positive modes with an Orbitrap analyzer under the same conditions as those used in a previous study [57].

### MaxQuant database search and protein quantification

Raw MS files were processed with MaxQuant (Version 1.5.6.0). LFQ was performed using a label-free, intensity-based absolute quantification approach.

Significant differences in protein levels were defined for proteins with different expression multiples (ratio A/B > 1.5 or ratio A/B < 0.66). Subsequently, we performed Gene Ontology (GO) and Kyoto Encyclopedia of Genes and Genomes (KEGG) pathway analyses, as well as protein-protein interaction analysis.

### ELISA

The levels of Ach (E20535), acetylcholine esterase (AchE, E2148), choline acetyltransferase (ChAT, E21422), Gln (E92310M), GABA (E20434M), iso-Hyd (E20542M), 5-HT (E20435M), NA (E91467M), HIS (E20452M) and 5-HIAA (E91494M) in the hippocampus were measured using the relevant ELISA kits according to the corresponding manufacturer’s instructions. The levels of Aβ1-42 (E20118), MDA (E20347M), CAT (E21414), SOD (E20348M), ROS (E20634) and GSH-Px (E20584) in the hippocampus and serum were measured using the relevant ELISA kits according to the corresponding manufacturer’s instructions (Shanghai Yuanye Biological Technology Co. Ltd., Shanghai, China).

### Statistical analysis

Data are expressed as the mean ± standard deviation (S.D.). Differences were determined by one-way analysis of variance followed by post-hoc multiple comparisons (Dunn’s test) using SPSS 16.0 software (IBM Corporation, Armonk, USA). Statistical significance was declared for *p*-values less than 0.05.

## Supplementary Material

Supplementary Figures

Supplementary Table 1
