# Supplementary material for: The neuroprotection of deproteinized calf blood extractives injection against Alzheimer's disease via regulation of Nrf-2 signaling
Source: Aging (Albany NY). 2021 Mar 26;13(8):11150–69. doi: 10.18632/aging.202776 (PMC8109110; doi:10.18632/aging.202776)
Supplement: Supplementary Figures [file aging-13-202776-s001.pdf]

SUPPLEMENTARY FIGURES

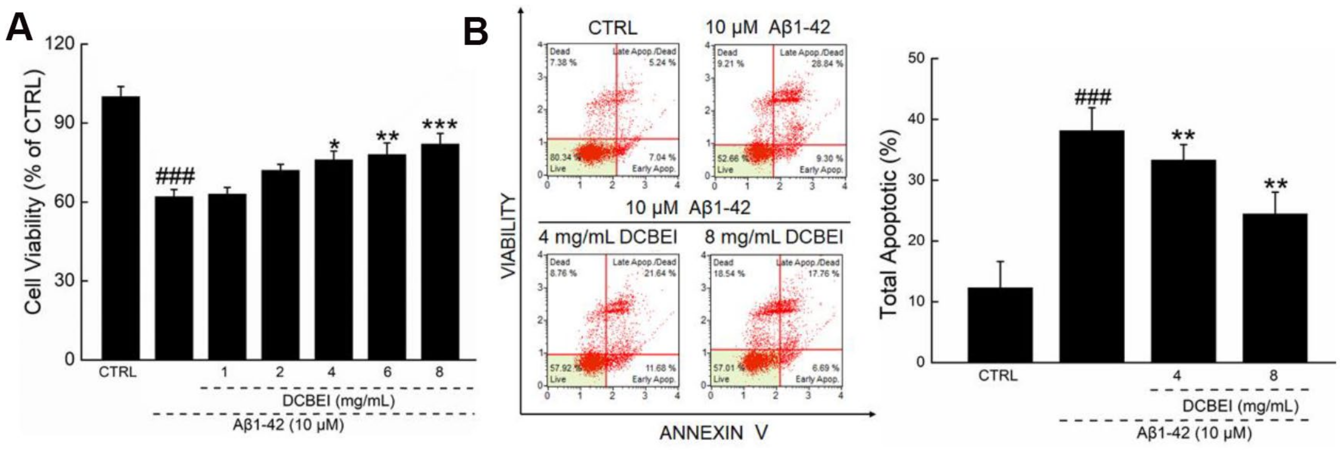

Supplementary Figure 1. DCBEI protects U251 cells against Aβ$_{1-42}$-induced damage. U251 cells were pre-incubated with DCBEI (4 mg/mL to 8 mg/mL) for 3 h, and co-treated with Aβ$_{1-42}$ for a further 24 h. (**A**) DCBEI increased cell viability. (**B**) DCBEI inhibited apoptosis. Data are expressed as a percentage of corresponding control cells and means ± S.D. (n = 6). ### *P* < 0.001 vs. CTRL, *. *P* < 0.05, * *P* < 0.01 and *** *P* < 0.001 vs. Aβ$_{1-42}$-treated cells.

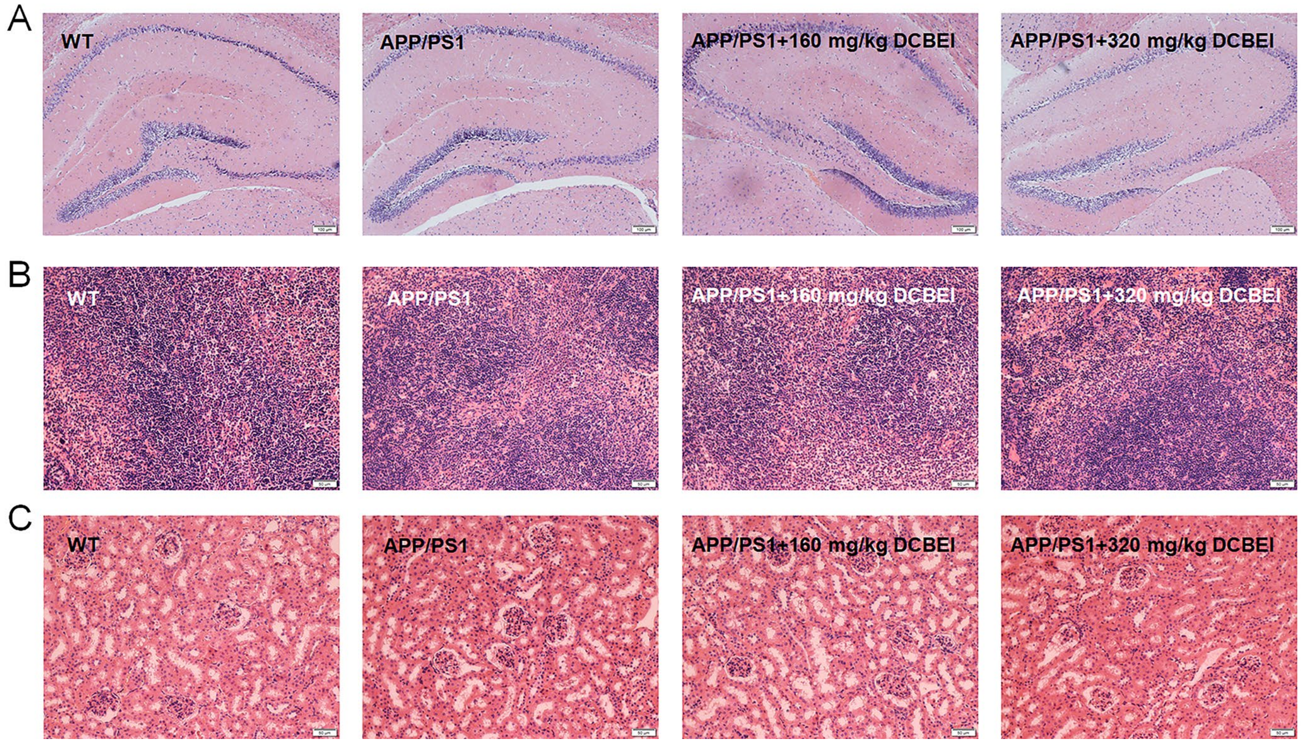

Supplementary Figure 2. Histopathological analysis via hematoxylin and eosin (H&E) staining. No significant changes on (**A**) brain tissues (100×) (Scale bar: 100 μm), (**B**) spleen tissues (200×) (Scale bar: 50 μm) and (**C**) kidney tissues (200×) (scale bar: 50 μm) were noted among all experimental groups including WT, APP/PS1, and DCBEI-treated APP/PS1 mice (n=3).

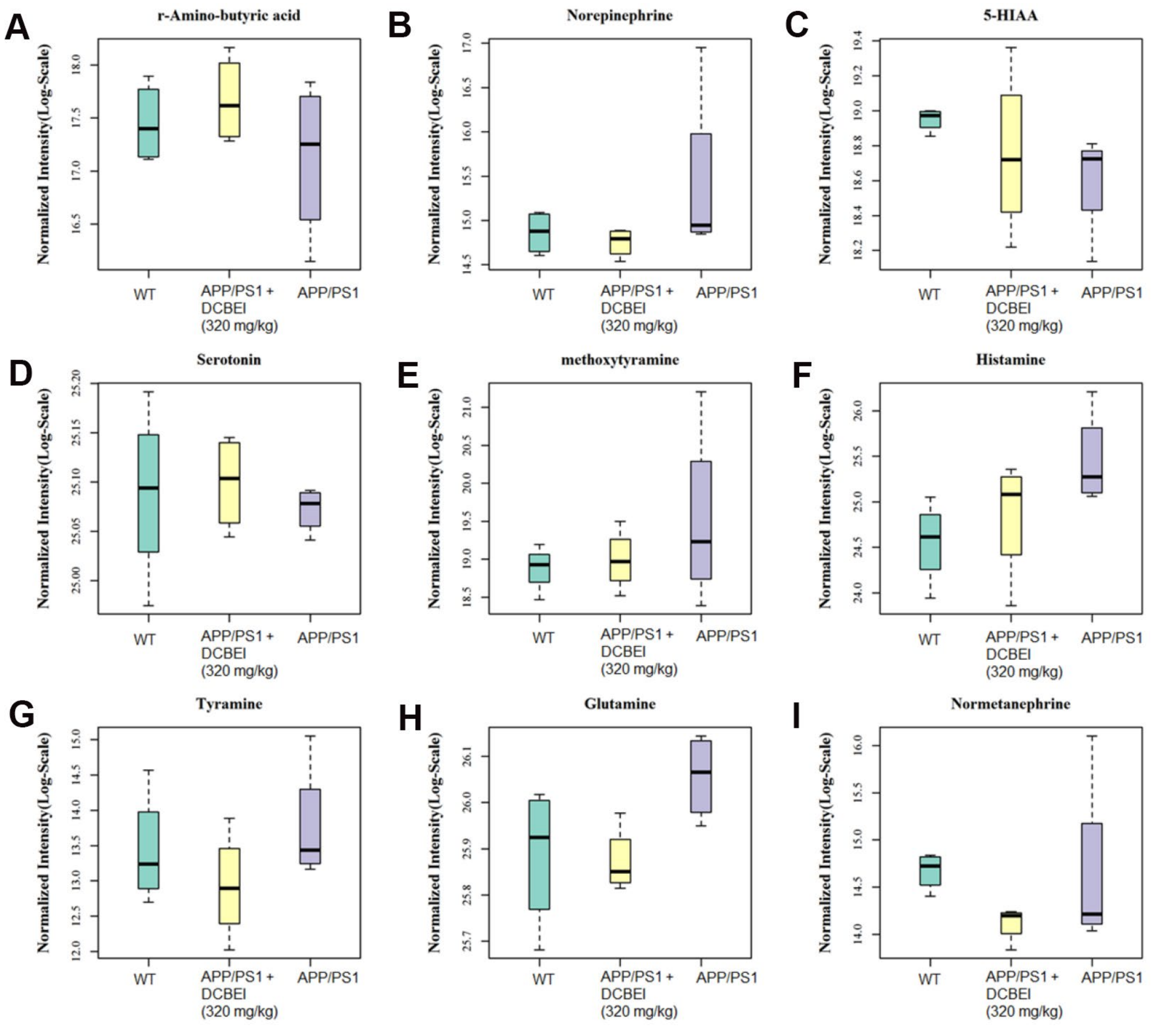

Supplementary Figure 3. DCBEI regulates neurotransmitter levels in serum analyzing by metabonomics via LC-MS/MS in APP/PS1 mice (n=3) including (**A**) r-amino-butyric acid, (**B**) norepinephrine, (**C**) 5-HIAA, (**D**) serotonin, (**E**) methoxytyramine, (**F**) histamine, (**G**) tyramine, (**H**) glutamine, and (**I**) normetanephrine.

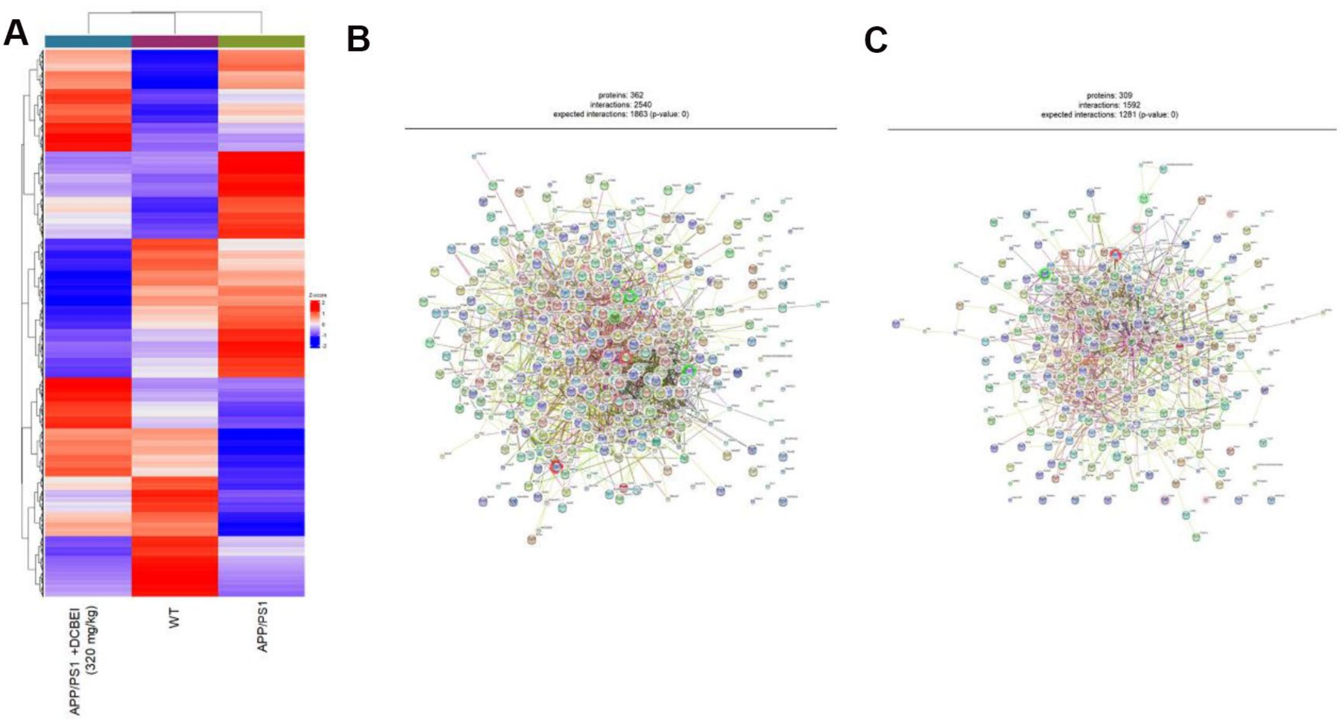

Supplementary Figure 4. DCBEI broadly regulates protein expression analyzing by proteomics. (**A**) The list of differentially expressed proteins among each experimental group. Protein–protein interaction network of (**B**) WT vs. APP/PS1 group and (**C**) APP/PS1 vs. DCBEI group analysis via STRINGdb.

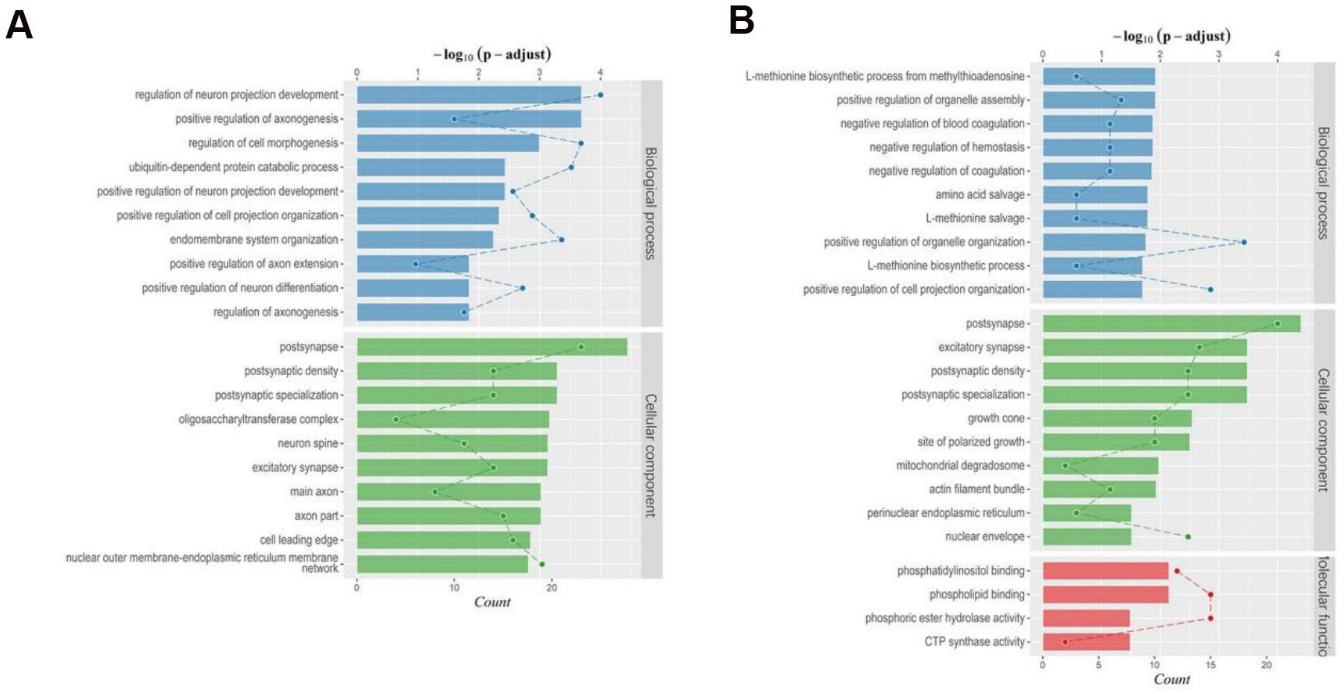

Supplementary Figure 5. GO enrichment analysis of (**A**) WT vs. APP/PS1 group and (**B**) APP/PS1 vs. DCBEI group.
