# Supplementary material for: The neuroprotection of deproteinized calf blood extractives injection against Alzheimer's disease via regulation of Nrf-2 signaling
Source: Aging (Albany NY). 2021 Mar 26;13(8):11150–69. doi: 10.18632/aging.202776 (PMC8109110; doi:10.18632/aging.202776)
Supplement: Supplementary Table 1 [file aging-13-202776-s002.pdf]

Supplementary Table 1. Antibodies used in western blot.

Description	Number	Source	Dilution
B-cell lymphoma-2 (Bcl-2)	ab7973	Abcam, Cambridge, MA, USA	1:1000
Bcl-XL	ab32370	Abcam, Cambridge, MA, USA	1:1000
Bcl-2 associated X protein (Bax)	ab7977	Abcam, Cambridge, MA, USA	1:2000
Bid	ab32060	Abcam, Cambridge, MA, USA	1:1000
Bad	ab32445	Abcam, Cambridge, MA, USA	1:2000
Cleaved poly (ADP-ribose) polymerase (Cleaved-PARP)	ab32064	Abcam, Cambridge, MA, USA	1:2000
Glyceraldehyde-3-phosphate dehydrogenase (GAPDH)	bs0755R	Bioss Inc. China	1:1000
Calpain-1	bs1099R	Bioss Inc. China	1:1000
P-RSK1 p90	ab32413	Abcam, Cambridge, MA, USA	1:2000
RSK1 p90	ab32526	Abcam, Cambridge, MA, USA	1:2000
P-Drp1	bs12701R	Bioss Inc. China	1:1000
Drp1	ab184247	Abcam, Cambridge, MA, USA	1:1000
heme oxygenase-(HO-1) 1	ab137749	Abcam, Cambridge, MA, USA	1:1000
superoxide dismutase 1 (SOD 1)	bs10216R	Bioss Inc. China	1:1000
superoxide dismutase 2 (SOD 2)	bs20667R	Bioss Inc. China	1:1000
cysteine ligase catalytic subunit (GCLC)	bs23393R	Bioss Inc. China	1:1000
GCLM	bs23361R	Bioss Inc. China	1:1000
NAD(P)H/quinone oxidoreductase 1 (NQO1)	bs-3184R	Bioss Inc. China	1:1000
C-Maf	ab77071	Abcam, Cambridge, MA, USA	1:1000
PKC-α	ab32376	Abcam, Cambridge, MA, USA	1:2000
Nuclear factor-erythroid 2 related factor 2 (Nrf-2)	ab137550	Abcam, Cambridge, MA, USA	1:1000
P-AKT (Ser473)	ab200195	Abcam, Cambridge, MA, USA	1:2000
Protein kinase B (AKT)	4060s	Santa Cruz Biotechnology, USA	1:2000
P-mTOR (S2448)	ab109268	Abcam, Cambridge, MA, USA	1:5000
mTOR	ab83495	Abcam, Cambridge, MA, USA	1:2000
P-JNK1/2/3	ab124956	Abcam, Cambridge, MA, USA	1:2000
c-JunN-terminalkinase (JNK)	ab112501	Abcam, Cambridge, MA, USA	1:2000
P-PTEN(Ser380)	bs3350R	Bioss Inc. China	1:1000
PTEN	bs0686R	Bioss Inc. China	1:1000
P-P38	ab4822	Abcam, Cambridge, MA, USA	1:1000
P38	ab75952	Abcam, Cambridge, MA, USA	1:2000
P-ERK	4370	Santa Cruz Biotechnology, USA	1:2000
Extra Cell ular signal-regulated kinase (ERK)	ab36991	Abcam, Cambridge, MA, USA	1:2000
Cytochrome C (Cyto C)	ab110325	Abcam, Cambridge, MA, USA	1:1000
β- Actin	ab8229	Abcam, Cambridge, MA, USA	1:500
Lamin B	bs-1840R	Bioss Inc. China	1:1000
